# Rosmarinic Acid Methyl Ester Inhibits LPS-Induced NO Production via Suppression of MyD88- Dependent and -Independent Pathways and Induction of HO-1 in RAW 264.7 Cells

**DOI:** 10.3390/molecules21081083

**Published:** 2016-08-18

**Authors:** Yangkang So, Seung Young Lee, Ah-Reum Han, Jin-Baek Kim, Hye Gwang Jeong, Chang Hyun Jin

**Affiliations:** 1 Advanced Radiation Technology Institute, Korea Atomic Energy Research Institute, Jeongeup-si, Jeollabuk-do 56212, Korea; yangkang@kaeri.re.kr (Y.S.); arhan@kaeri.re.kr (A.-R.H.); jbkim74@kaeri.re.kr (J.-B.K.); 2 College of Pharmacy, Chungnam National University, Daejeon, Chungcheongnam-do 34134, Korea; hgjeong@cnu.ac.kr; 3 Freshwater Bioresources Utilization Division, Nakdonggang National Institute of Biological Resources, Sangju-si, Gyeongsangbuk-do 37242, Korea; nplsy001@nnibr.re.kr

**Keywords:** *Perilla frutescens*, anti-inflammation, rosmarinic acid methyl ester, MyD88-dependent and -independent pathways, heme oxygenase-1

## Abstract

In this study, we investigated the anti-inflammatory effect of rosmarinic acid methyl ester (RAME) isolated from a mutant cultivar of *Perilla frutescens* (L.) Britton. We found that RAME inhibits lipopolysaccharide (LPS)-induced nitric oxide (NO) production, with an IC_50_ of 14.25 µM, in RAW 264.7 cells. RAME inhibited the LPS-induced expression of pro-inflammatory cytokines including interleukin (IL)-1β, IL-6, IL-10, monocyte chemoattractant protein-1, interferon-β, and inducible nitric oxide synthase (iNOS). Moreover, RAME suppressed the activation of nuclear factor kappa B. These results suggest that the downregulation of iNOS expression by RAME was due to myeloid differentiation primary response gene 88 (MyD88)-dependent and -independent pathways. Furthermore, RAME induced the expression of heme oxygenase-1 (HO-1) through activation of nuclear factor-erythroid 2-related factor 2. Treatment with tin protoporphyrin, an inhibitor of HO-1, reversed the RAME-induced suppression of NO production. Taken together, RAME isolated from *P. frutescens* inhibited NO production in LPS-treated RAW 264.7 cells through simultaneous induction of HO-1 and inhibition of MyD88-dependent and -independent pathways.

## 1. Introduction

Inflammation, involved in the non-specific immune system, occurs in response to bodily injury and is required for the continuation of health in the presence of bacterial and viral infections [1]. Acute and chronic inflammation can prove harmful, contributing to the pathogenesis of a variety of diseases including inflammatory bowel disease, multiple sclerosis, atherosclerosis, arthritis, and asthma [2,3]. Macrophages play an important role in inflammatory diseases related to over-production of inflammatory mediators including interleukin (IL)-1β, IL-6, IL-10, monocyte chemoattractant protein (MCP)-1, tumor necrosis factor (TNF)-α reactive oxygen species (ROS), and nitric oxide (NO) [4,5]. Nitric oxide synthase (NOS), which is responsible for NO synthesis from l-arginine, has three isoforms: endothelial NOS (eNOS), neuronal NOS (nNOS), and inducible NOS (iNOS) [6,7]. Commonly, macrophages express iNOS in response to inflammatory mediators such as lipopolysaccharide (LPS) and interferon (IFN)-γ; however, eNOS and nNOS are expressed constitutively [8,9]. NO production mediates many diseases such as obesity [10], carcinogenesis [11], atherosclerosis [12], inflammation [12], and diabetes [12]. Therefore, a decrease in NO production by iNOS inhibition has therapeutic potential in various inflammatory conditions [13].

Heme oxygenase (HO) is an important enzyme responsible for maintaining intracellular heme levels. HO exists as three isoforms: HO-1, HO-2, and HO-3 [14]. HO-1 is expressed in cells at low levels under no stimulation [14]. However, its expression is inducible by a variety of stimuli including oxidative stress, LPS, melatonin, transforming growth factor-β1, and adenosine [15]. Enhancement of HO-1 expression plays an important role in cell protection [15], and HO-1 has anti-inflammatory activity, including inhibiting NO production [16]. Its expression is regulated at the transcriptional level and is related to the transcription factor nuclear factor-erythroid 2-related factor 2 (Nrf2). Nrf2 is an upstream transcription factor that modulates phase ІІ enzyme activity [17]. Under physiological conditions, Nrf2 is sequestered by binding to Kelch-like ECH associated protein 1 (Keap1). However, upon stimulation, Nrf2 is released from Keap1 and translocated to the nucleus to induce HO-1 expression [18].

**Figure 1:**
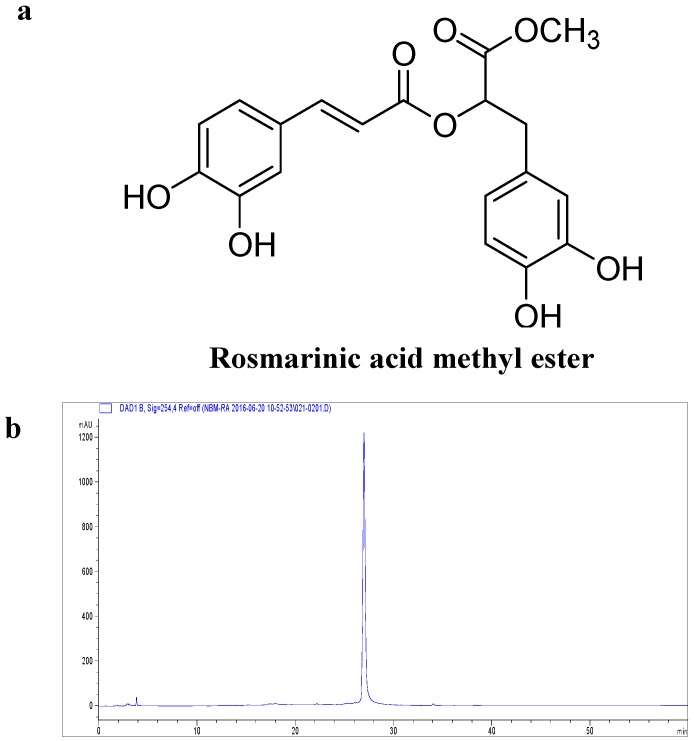

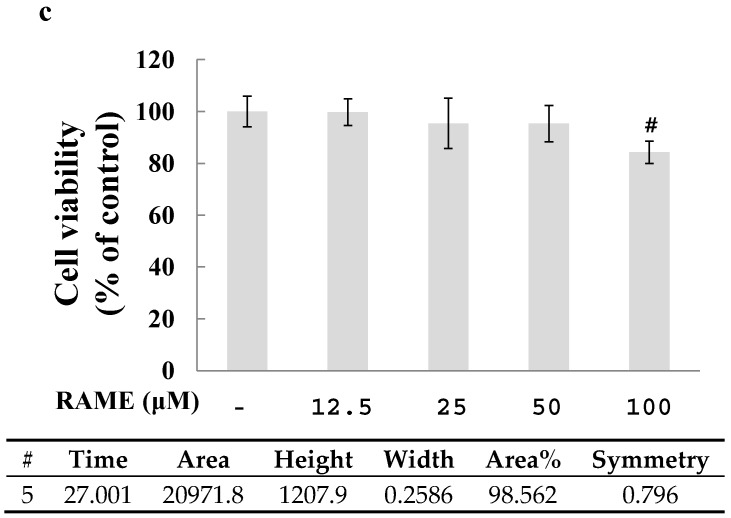
Chemical structure of RAME and its effects on cell viability. (**a**) Chemical structure of RAME; (**b**) Purity of RAME from a mutant cultivar of *P. frutescens* measured by HPLC analysis; (**c**) Cell viability. # *p* < 0.05 vs. control.

*Perilla frutescens* (L.) Britton is an edible plant and a popular and frequently used garnish in some Asian countries such as China, Japan, and Korea. *P. frutescens* has been shown previously to have detoxicant, antitussive, antibiotic, and antipyretic effects, and it is utilized in traditional medicine [19,20]. The biological activity of *P. frutescens* has been well investigated. Previous studies have described the qualitative and quantitative presence of flavonoids and phenolic acids such as rosmarinic acid, catechin, apigenin, luteolin, caffeic acid, and ferulic acid in *P. frutescens* [21,22]. Moreover, it also contains rosmarinic acid methyl ester (RAME, Figure 1a), which exerts superoxide scavenging [23], anti-allergic [24], and anti-microbial [25] activities, as well as inhibits pro-inflammatory cytokines in the lung [26]. However, the mechanism underlying the anti-inflammatory effects of RAME has not been fully defined. Therefore, the aim of the present study was to evaluate the mechanism of the anti-inflammatory effects of RAME in LPS-treated RAW 264.7 cells.

## 2. Results

### 2.1. Effect of RAME on Cytotoxicity

As shown in Figure 1b, the purity of RAME was 98.562% as determined by high performance liquid chromatography (HPLC). RAW 264.7 cells were treated with various concentrations of RAME for 24 h, and cytotoxicity was assessed using the EZ-Cytox cell viability assay kit (Figure 1c. RAME did not exhibit cytotoxicity at 50 µM, but at 100 µM, it inhibited cell viability. Based on these results, RAME doses of 12.5, 25, and 50 µM were used in this study.

### 2.2. Effect of RAME on NO Production and iNOS Protein Expression Levels

**Figure 2:**
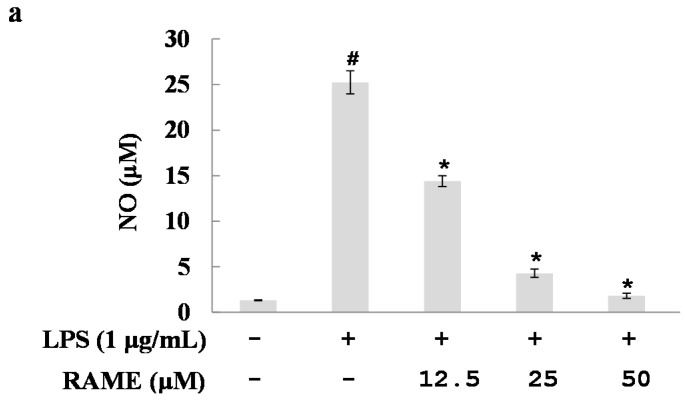

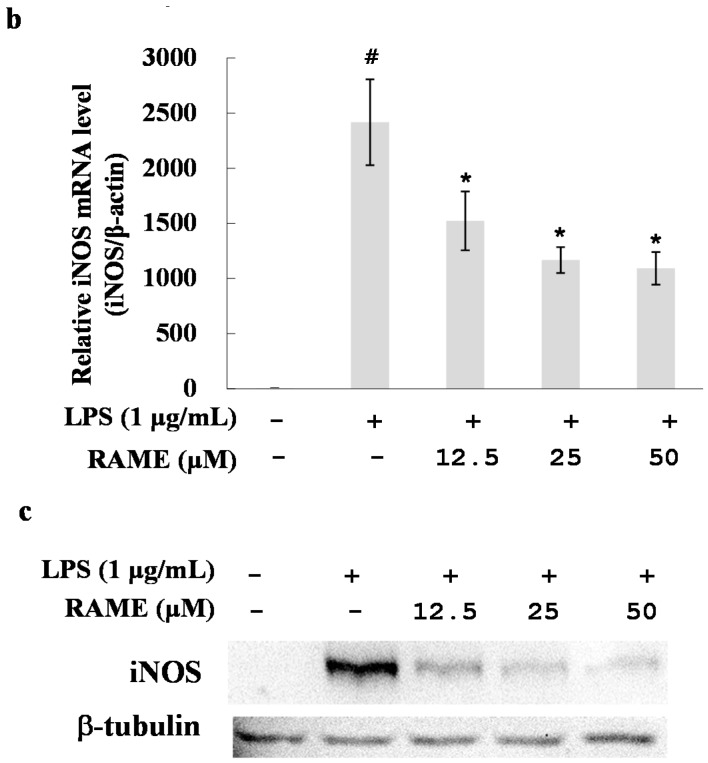
Effects of RAME on NO production and iNOS expression levels in RAW 264.7 cells. RAW 264.7 cells were treated with various concentrations of RAME for 2 h prior to incubation with LPS (1 μg/mL) for 18 h. (**a**) Inhibition of NO production; (**b**) mRNA expression levels of iNOS measured by real-time PCR; (**c**) Protein expression levels of iNOS measured by Western blot. # *p* < 0.05 vs. control group, * *p* < 0.05 vs. LPS-treated.

To evaluate whether RAME has anti-inflammatory properties, we investigated the effect of RAME on NO production in LPS-treated RAW 264.7 cells. The levels of NO production in the cell culture supernatant were determined using the Griess reagent. The increased NO production by LPS was significantly suppressed by pretreatment with RAME in a dose-dependent manner (Figure 2a). The IC_50_ value of NO inhibition was 14.25 µM. iNOS is responsible for NO production during inflammation [5].

We examined the effect of RAME on iNOS expression in LPS-treated RAW 264.7 cells; LPS treatment resulted in increased iNOS expression. The mRNA levels of iNOS were suppressed by treatment with RAME as measured by quantitative real-time polymerase chain reaction (PCR) (Figure 2b). Moreover, western blot analysis showed that the protein levels of iNOS were suppressed by RAME in a dose-dependent manner (Figure 2c). These results suggest that NO production was inhibited by RAME in LPS-treated RAW 264.7 cells via suppression of iNOS expression.

### 2.3. Effect of RAME on NF-κB Activation in LPS-Treated RAW 264.7 Cells

**Figure 3:**
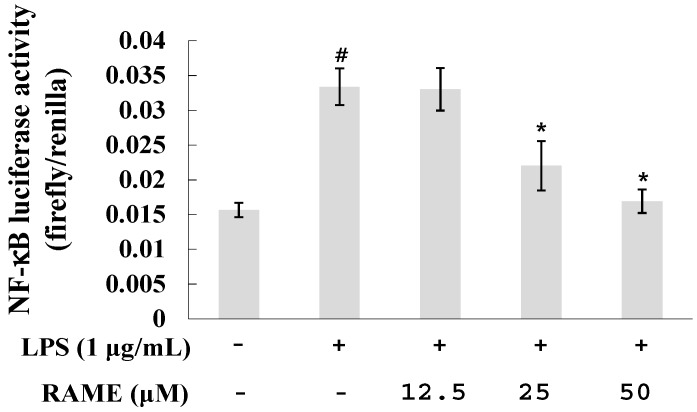
Effects of RAME on the NF-κB pathway. # *p* < 0.05 vs. control group , * *p* < 0.05 vs. LPS-treated group.

LPS is generally activated by a myeloid differentiation primary response gene 88 (MyD88)-dependent pathway via Toll-like receptor 4 (TLR4). Activation of this MyD88-dependent pathway induces translocation of NF-κB to the nucleus for production of pro-inflammatory mediators including ROS, NO, and iNOS [27]. NF-κB is an important transcription factor involved in iNOS expression [27]. To investigate whether the effect of RAME on iNOS expression was due to inhibition of NF-κB activation, we examined the transcriptional activity of NF-κB using a luciferase assay (Figure 3).

NF-κB activity was increased in LPS-treated RAW 264.7 cells. However, its activity was reduced by treatment with RAME in a dose-dependent manner. This result indicates that RAME inhibited iNOS expression via suppression of NF-κB activation.

### 2.4. Effect of RAME on the Expression of Pro-Inflammatory Cytokines in LPS-Treated RAW 264.7 Cells

**Figure 4:**
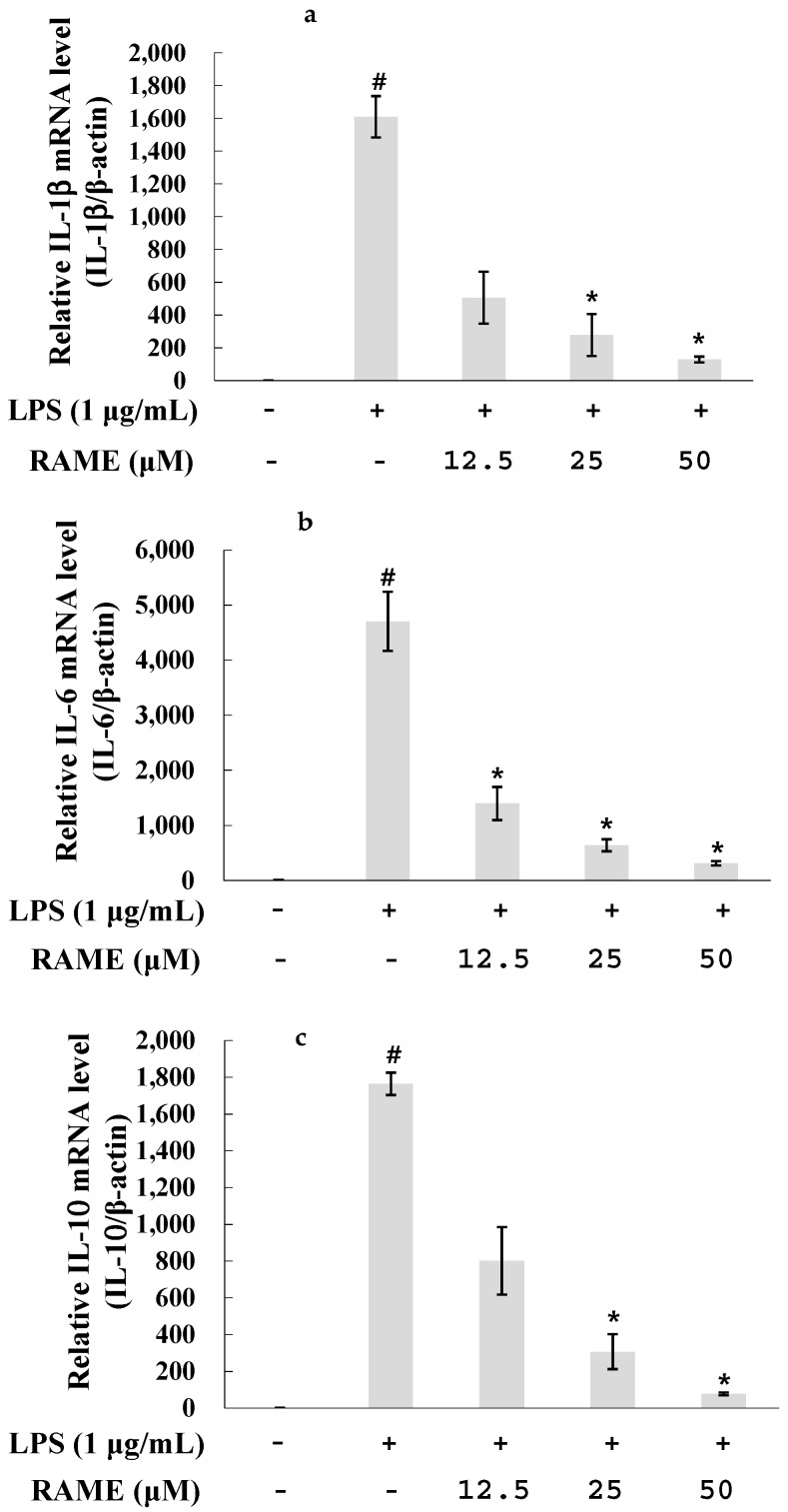
Effects of RAME on IL-1β, IL-10, and IL-6 mRNA expression levels in LPS-treated RAW 264.7 cells. The expression level of pro-inflammatory cytokine genes were measured by real-time PCR. RAW 264.7 cells were treated RAME (12.5, 25, and 50 µM) with LPS (1 µg/mL) and incubated for an additional 18 h. mRNA expression level of genes was normalized with β-actin. (**a**) IL-1β mRNA expression level; (**b**) IL-6 mRNA expression level; (**c**) IL-10 RNA expression level. The results are presented as the means ± SDs of three replicates of one representative experiment. # *p* < 0.05 vs. control group , * *p* < 0.05 vs. LPS-treated group.

NF-κB activation is related to overproduction of pro-inflammatory cytokines including IL-1β, IL-6, IL-10, MCP-1, ROS, and NO [4,5]. To determine the effects of RAME on these pro-inflammatory cytokines, RAW 264.7 cells were incubated with various concentrations of RAME in the presence or absence of LPS for 18 h. The mRNA levels of IL-1β, IL-6, and IL-10 were measured by real-time PCR. As shown in Figure 4a–c, the mRNA levels of these pro-inflammatory cytokines were significantly reduced by treatment with RAME in a dose-dependent manner. Therefore, RAME-mediated suppression of NF-κB was confirmed.

### 2.5. Effect of RAME on the IFN-β Pathway in LPS-Treated RAW 264.7 Cells

**Figure 5:**
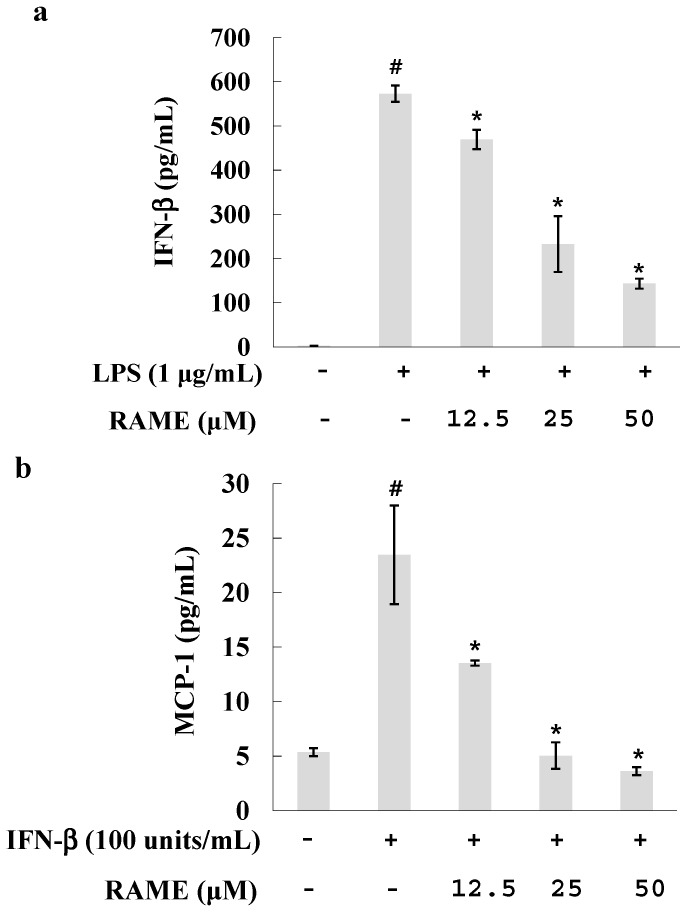
Effects of RAME on the IFN-β pathway. (**a**) RAW 264.7 cells were treated with RAME for 2 h prior to incubation with LPS (1 μg/mL) for 4 h. IFN-β levels were detected in the culture supernatant using an ELISA kit. # *p* < 0.05 vs. control group, * *p* < 0.05 vs. LPS-treated group; (**b**) RAW 264.7 cells were treated with various concentrations of RAME for 2 h prior to incubation with IFN-β (100 units/mL) for 8 h. MCP-1 levels were detected in the culture supernatant using an ELISA kit. # *p* < 0.05 vs. control group , * *p* < 0.05 vs. IFN-β-treated group.

IFN-β is highly upregulated and then secreted in response to LPS [28]. Recent reports have implicated IFN-α/β as an autocrine/paracrine signal crucial for the induction of iNOS [28], and IFN-β exhibited synergistic effects with LPS in RAW 264.7 cells, resulting in faster and greater production of NO [16]. To investigate whether RAME affects the IFN-β pathway, we measured IFN-β levels using an enzyme-linked immunosorbent assay (ELISA) kit. LPS-treated RAW 264.7 cells were pretreated with various concentrations of RAME, and IFN-β production was suppressed by RAME in a dose-dependent manner (Figure 5a). This result suggests that RAME suppressed IFN-β levels, which could be related to NO production.

Furthermore, we investigated whether RAME could modulate the downstream pathway of IFN-β. In the MyD88-independent pathway of LPS activation, IFN-β exerts its effects through the IFN-β receptor, which itself does not induce NO production [16]. However, IFN-β can induce production of the pro-inflammatory cytokine MCP-1 in RAW 264.7 cells [16]. Therefore, we examined the effect of RAME on MCP-1 levels in IFN-β-treated RAW 264.7 cells. RAW 264.7 cells were treated with various concentrations of RAME together with IFN-β (100 units/mL). MCP-1 levels were increased by IFN-β but suppressed by RAME in a dose-dependent manner (Figure 5b). These results suggest that RAME mediated the upstream and downstream pathways of IFN-β production in LPS-treated RAW 264.7 cells.

### 2.6. Effect of RAME on HO-1 Induction and Nrf2 Activation

**Figure 6:**
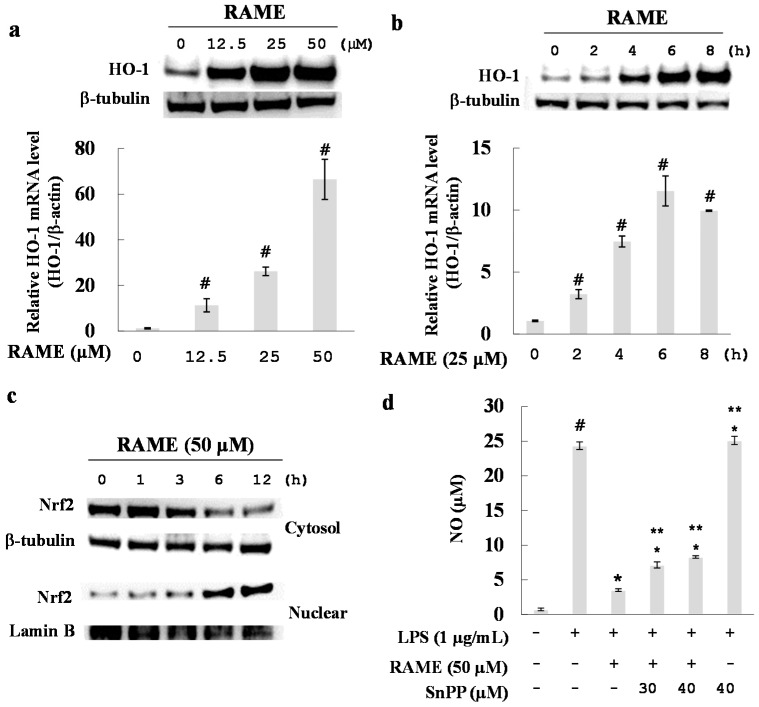
Effects of RAME on HO-1 expression. (**a**) RAW 264.7 cells were incubated with various concentrations of RAME for 6 h. The HO-1 mRNA expression level was determined by quantitative real-time PCR. HO-1 protein expression levels were measured by western blot analysis. # *p* < 0.05 vs. control group; (**b**) RAW 264.7 cells were treated with 25 μM RAME for 0, 2, 4, 6 and 8 h. Total RNA was isolated and used to measure HO-1 mRNA expression levels by quantitative real-time PCR. # *p* < 0.05 vs. control group; (**c**) Cytosolic and nuclear protein fractions were extracted after treatment with RAME (50 μM) for 0, 1, 3, 6 and 12 h. Protein levels were normalized to those of β-tubulin and lamin B; (**d**) RAW 264.7 cells were treated with either RAME or RAME plus SnPP, an HO-1 inhibitor, for 2 h prior to incubation with LPS (1 μg/mL) for 18 h. # *p* < 0.05 vs. control group, * *p* < 0.05 vs. LPS-treated group and ** *p* < 0.05 vs. LPS + RAME group.

HO-1 plays a critical role in the regulation of inflammation as it protects macrophages from ROS and helps inhibit NO production in LPS-treated RAW 264.7 cells [29]. We investigated the effect of RAME on HO-1 induction. RAME led to an increase in the mRNA and protein expression levels of HO-1. As shown in Figure 6b, RAME increased HO-1 expression in a dose-dependent manner, with a maximal increase after 6 h of treatment (Figure 6a).

Generally, the transcription factor Nrf2 translocates to the nucleus to induce expression of HO-1 in RAW 264.7 cells [18]. We found that RAME increased the translocation of Nrf2 to the nucleus (Figure 6c).

To investigate whether HO-1 induction is responsible for the RAME-mediated inhibition of LPS-induced NO production, cells were treated with LPS and RAME in the presence of tin protoporphyrin (SnPP), a specific inhibitor of HO-1, and NO levels were measured. SnPP alone did not significantly affect LPS-stimulated NO production; however, it restored RAME-mediated suppression of NO production in a dose-dependent manner (Figure 6d). These results suggest that HO-1 induced by RAME was involved in the RAME-induced inhibition of NO production.

## 3. Discussion

In previous studies, several compounds isolated from *P. frutescens* were evaluated for their potential biological activities [30,31]. The anti-inflammatory effect of rosmarinic acid (RA) has been reported in several papers [21,32,33]. RA had anti-inflammatory activity through the suppression of activation of ERK, JNK, p38, and NF-κB [32]. Furthermore, RA inhibited expression of cyclooxygenase-2 (COX-2) in LPS-treated RAW 264.7 cells [33]. Even if RAME had similar anti-inflammatory effect with RA such as suppression of NF-κB activation, RAME did not affect the expression of COX-2 in LPS-treated RAW 264.7 cells (data not shown). Therefore, those two compounds have different anti-inflammatory mechanisms. In this study, we investigated a mutant cultivar of *P. frutescens* obtained via mutagenesis by γ-rays [34]. Specifically, we examined the anti-inflammatory mechanism of RAME isolated from this mutant in LPS-treated RAW 264.7 cells. The purity of the isolated RAME was 98.562%, as determined with HPLC analysis (Figure 1b). The anti-inflammatory activity of RAME was determined under non-toxic concentrations (Figure 1c). RAME suppressed NO production by inhibiting iNOS expression in LPS-treated RAW 264.7 cells (Figure 2). Therefore, we suggest that RAME exerts important anti-inflammatory activity by inhibiting iNOS expression.

LPS of gram-negative bacteria is recognized by TLR4. LPS activates two pathways: (1) the MyD88-dependent pathway and (2) the TIR domain-containing adaptor protein (TRIF)-dependent pathway [35]. Upon activation, MyD88 recruits IL-1 receptor-associated kinase (IRAK) family members, including IRAK1, IRAK2, IRAK4, and IRAK-M. IRAK4 is sequentially phosphorylated, leading to its dissociation from MyD88, which activates the TNF receptor-associated factor 6 (TRAF6) [36]. TRAF6 subsequently activates transforming growth factor-β-activated protein kinase 1 (TAK1), which is a member of the MAP kinase family [37]. TAK1 activates the IKK complex and MAP kinases, resulting in the translocation of NF-κB and activator protein-1 to the nucleus. Translocation of NF-κB results in the production of inflammatory cytokines [36]. Much research has demonstrated that inhibiting the production of pro-inflammatory mediators via the NF-κB and MAPK pathways suppresses the inflammatory response [38,39,40]. Our results suggest that RAME suppresses iNOS expression via the NF-κB pathway (Figure 3). LPS can induce pro-inflammatory cytokines, including IL-6, IL-10, IL-1β, and MCP-1, in macrophages [41,42]. We showed that RAME significantly inhibited the production of IL-6, IL-10, and IL-1β in LPS-treated RAW 264.7 cells (Figure 4a–c). These findings confirm RAME-mediated inhibition of NO production via the MyD88-dependent pathway by suppression of anti-inflammatory cytokines and NF-κB activation.

The TRIF-dependent pathway is important in IFN-β production. TRIF interacts with TANK-binding kinase-1, which together with IKKi, mediates the phosphorylation of interferon regulatory transcription factor 3. Moreover, TRIF activates NF-κB and MAPK through TRAF6 and receptor interacting protein 1. These pathways are required for the induction of IFN-β [35]. IFN-β does not exert its effect through TLR4 but rather through the IFN-β receptor. IFN-β cannot induce NO production alone but rather acts in synergy with LPS [16]. When we treated RAW 264.7 cells with LPS and IFN-β, NO was produced at a faster rate and higher levels than when treated with LPS alone. IFN-β alone induced the production of MCP-1 in RAW 264.7 cells [16]. This increase was reduced by treatment with RAME (Figure 5b). These findings indicate that RAME suppressed the upstream and downstream pathways of IFN-β in RAW 264.7 cells. Moreover, the RAME-mediated inhibition of IFN-β may be involved in the reduction of NO production.

LPS rapidly increases ROS levels in macrophages and directly damages DNA and induces excessive inflammatory factors and cytokines [41]. HO-1 is important for the protection of macrophages from ROS and has recently become a popular target for the production of antioxidants. Nrf2 is an upstream mediator of antioxidant response element-dependent phase ІІ enzyme expression, including HO-1 [43]. An inactivated form of Nrf2 exists in the cytoplasm, bound to Kelch-like ECH-associated protein 1 (Keap1). However, upon stimulation, Nrf2 dissociates from Keap1 and translocates to the nucleus to induce HO-1 expression [43]. We found that HO-1 mRNA and protein expression levels were increased by RAME via translocation of Nrf2 to the nucleus (Figure 6a–c). Furthermore, the inhibitory effect of RAME on NO production in LPS-treated RAW 264.7 cells was attenuated by treatment with SnPP, a specific HO-1 inhibitor, in a dose dependent manner (Figure 6d). Taken together, these results suggest that RAME-induced HO-1 expression was caused by the translocation of Nrf2, which may be related to NO production. The exact mechanism of RAME-induced Nrf2 translocation should be investigated in future studies.

**Figure 7:**
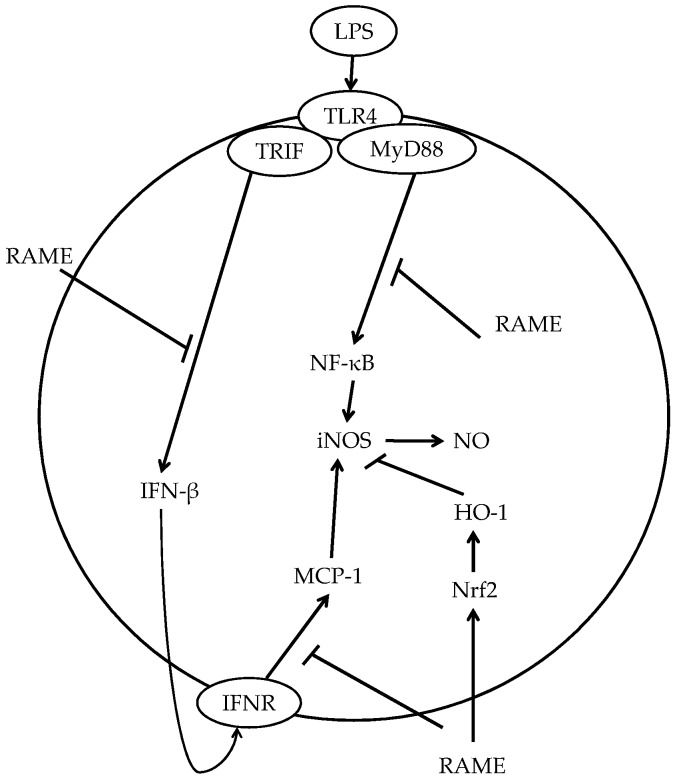
Possible model for the inhibition of iNOS or NO production by RAME in LPS-treated RAW 264.7 cells.

These results suggest that RAME had anti-inflammatory activities through the MyD88-dependent pathway and -independent pathways. At this point, there is a possibility that RAME could interrupt LPS/TLR4-mediated signaling [44] directly. However, RAME also affected TLR4-independent pathways [15] for anti-inflammatory activity. We found that RAME induced HO-1 expression for inhibition of NO production via the TLR4-independent pathway. Furthermore, RAME inhibited MCP-1 production in IFN-β-treated RAW 264.7 cells (Figure 7). The production of MCP-1 was induced via IFN-β/IFNR pathway in IFN-β-treated RAW 264.7 cells [16]. Therefore, RAME suppressed NO production via additional anti-inflammatory pathway except TLR4. Based on these results, RAME should be considered a potential anti-inflammatory agent for the treatment of inflammatory diseases.

## 4. Materials and Methods

### 4.1. General Procedures

The 1D NMR experiment was performed on a JNM-ECA 500MHz NMR instrument (JEOL Ltd., Tokyo, Japan). Thin-layer chromatographic (TLC) analysis was performed on Kieselgel 60 F254 (Merck, Darmstadt, Germany), with visualization under UV light (254 and 365 nm) and 10% (*v*/*v*) sulfuric acid spray followed by heating (200 °C, 2 min). YMC Gel ODS-A (12 nm, S-150 μm; YMC Co., Kyoto, Japan), and Sephadex LH-20 (Pharmacia Co., Uppsala, Sweden) were used for column chromatography (CC). Analytical HPLC was carried out on an Agilent 1100 series system (Agilent Technologies Co., Santa Clara, CA, USA) equipped with a YMC-Triart C18 column (5 μm, 250 mm × 4.6 mm, YMC Co.).

### 4.2. Materials

The leaves of the mutant cultivar of *P. frutescens* were collected by the radiation breeding research team of the Korea Atomic Energy Research Institute [32]. The leaves were air-dried, pulverized, and stored at 4 °C before extraction. Dulbecco’s modified Eagle’s medium (DMEM), fetal bovine serum (FBS), and penicillin-streptomycin were purchased from Hyclone (Logan, UT, USA). LPS, dimethyl sulfoxide (DMSO), Griess reagent, and protease inhibitor cocktail were purchased from Sigma-Aldrich (St. Louis, MO, USA). Opti-MEMI, goat anti-rabbit IgG horseradish peroxidase (HRP)-conjugated antibody, and Lipofectamine 2000 were purchased from Invitrogen (Carlsbad, CA, USA). The HO-1 inhibitor SnPP was purchased from Porphyrin Products Inc. (Logan, UT, USA). The RNeasy kit was purchased from QIAGEN (Valencia, CA, USA), the EZ-Cytox Cell Viability assay kit from DAEIL Lab (Seoul, Korea), and the 1st Strand cDNA Synthesis kit and SYBR premix from Takara Bio Inc. (Kyoto, Japan). The NP40 cell lysis buffer was purchased from Biosource (San Jose, CA, USA). The rabbit anti-iNOS polyclonal antibody was purchased from Cell Signaling Technology (Danvers, MA, USA). The rabbit polyclonal antibodies against beta-tubulin, HO-1, Lamin B, and Nrf2 were purchased from Santa Cruz Biotechnology (Santa Cruz, CA, USA). The ELISA kits for MCP-1 and IFN-β were purchased from R&D System (Minneapolis, MN, USA).

### 4.3. Extraction and Isolation

Dried leaves (1.2 kg) of the mutant cultivar of *P. frutescens* were extracted twice with 100% methanol (10 L) at room temperature for 24 h, and the supernatant was evaporated under vacuum using an evaporator. The methanol extract (200 g) was dissolved in distilled water and partitioned three times using *n*-hexane, chloroform, ethyl acetate, and *n*-butanol. Evaporation of the solvent of the appropriate fraction under reduced pressure yielded the ethyl acetate extract (5 g), which was further fractionated on a reverse-phase (RP) silica gel column (YMC Gel ODS-A, 12 nm, S-150 μm; YMC Co.) and eluted using 35% methanol to give 10 fractions (PE1–PE10). PE6 (100 mg) was fractionated on a RP silica gel column and eluted using 50% methanol to give three fractions (PE61–PE63). PE63 (100 mg) was fractionated on a Sephadex LH-20 column and eluted using 80% methanol to yield *rosmarinic acid methyl ester* (RAME) as a yellowish powder (50 mg). The purity evaluation of RAME was determined by analytical HPLC (0 min, acetonitrile–water, 5:95; 45 min, 95:5; flow rate 1 mL/min, detector UV 254 nm). The *t*_R_ of RAME was 27.001 min, and its purity was 98.562%. Its structure was determined by analyses of its 1H-NMR data (Supplementary Materials Figure S1) as well as by comparison of its data with the published values [25]. ^1^H-NMR (CD_3_OD, 500 MHz): δ 7.53 (1H, d, *J* = 15.5 Hz, H-7), 7.03 (1H, d, *J* = 2.0 Hz, H-2), 6.95 (1H, dd, *J* = 8.0, 2.0 Hz, H-6), 6.77 (1H, d, *J* = 8.0 Hz, H-5), 6.69 (1H, d, *J* = 2.0 Hz, H-2′), 6.68 (1H, d, *J* = 8.0 Hz, H-5′), 6.55 (1H, dd, *J* = 8.0, 2.0 Hz, H-6′), 6.24 (1H, d, *J* = 16.0 Hz, H-8), 5.17 (1H, dd, *J* = 8.0, 5.4 Hz, H-8′), 3.67 (3H, s, OCH3), 3.03 (1H, dd, *J* = 14.2, 5.4 Hz, H-7′a), 3.00 (1H, dd, *J* = 14.2, 8.0 Hz, H-7′b).

### 4.4. Cell Culture

The RAW 264.7 cell line was obtained from ATCC. RAW 264.7 cells were cultured in DMEM supplemented with 10% FBS, 100 units/mL penicillin, and 100 µg/mL streptomycin and maintained in a humidified incubator at 37 °C in 5% carbon dioxide (CO_2_). 

### 4.5. Cytotoxicity Assay 

The EZ-Cytox cell viability assay kit was used to measure cell viability. The cells were cultured in a 96-well plate at a density of 2.0 × 10^5^ cells/mL for 24 h. RAME was dissolved in DMSO and incubated with the cells at various concentrations (12.5, 25, 50, and 100 µM) for an additional 24 h. After the incubation period, 10 μL solution of cell viability assay kit was added to each well and incubated for 4 h at 37 °C and 5% CO_2_. The index of cell viability was determined by measuring formazan production using a spectrophotometer (Benchmark Plus, Bio-Rad, Hercules, CA, USA) at an absorbance of 480 nm with a reference wavelength of 650 nm. The results are presented as means ± standard deviation (SD) of six replicates for one representative experiment.

### 4.6. Nitrite Assay 

The cells were cultured in a 96-well plate at a density of 2.0 × 10^5^ cells/mL for 24 h. After incubation, the cells were pretreated with various concentrations of RAME (12.5, 25, and 50 μM) for 2 h and then treated with LPS (1 μg/mL, dissolved in D.W.) for an additional 18 h. The culture supernatant (100 μL) was mixed with an equal volume of Griess reagent (100 μL) in a 96-well plate and incubated for 15 min at room temperature. The absorbance of each sample was measured at 540 nm using a spectrophotometer. The results are presented as means ± SD of six replicates for one representative experiment.

### 4.7. Western Blotting

The cells were cultured in a 100 mm culture dish at a density of 2.0 × 10^5^ cells/mL for 24 h. They were treated with RAME at various concentrations (12.5, 25, and 50 μM) for 2 h and subsequently with LPS (1 μg/mL) for 1 h (for Nrf2 protein expression), 6 h (for HO-1 protein expression), or 18 h (for iNOS protein expression). The cells were harvested and lysed using NP40 cell lysis buffer (with 1 mM phenylmethylsulfonyl fluoride and 1x protease inhibitor cocktail) for 30 min on ice, and the resulting cell extracts were centrifuged. Nuclear and cytosolic extracts were prepared using a NE-PER Nuclear and cytoplasmic extraction reagents (Pierce, Rockford, IL, USA). The protein concentration was quantified, and the proteins were separated on 10% running gels and transferred to nitrocellulose membranes. The membranes were incubated with primary antibodies (Nrf2, HO-1, iNOS, lamin B, and tubulin) at 4 °C overnight. The primary antibodies were diluted according to the manufacturer’s protocol. The membranes were washed four times with TBS-T for 15 min and then incubated with HRP-conjugated secondary antibodies for 2 h on a shaker at room temperature. The membranes were then washed again, and the proteins were detected using an enhanced chemiluminescence detection system.

### 4.8. Quantitative Real-Time Polymerase Chain Reaction

The cells were cultured in a 6-well plate at a density of 2.0 × 10^5^ cells/mL for 24 h. They were treated with RAME at various concentrations (12.5, 25 and 50 μM) for 2 h and were subsequently incubated with LPS (1 μg/mL) for an additional 18 h. Total RNA was isolated using the RNeasy kit according to the manufacturer’s protocol. The 1st Strand cDNA Synthesis kit was used for reverse transcription according to the manufacturer’s protocol. SYBR was used for real time PCR amplification for iNOS, HO-1, IL-10, IL-6, IL-1β and β-actin using the Chromo 4 RT-PCR detection system (Table 1).

### 4.9. Luciferase Assay

RAW 264.7 cells were cultured in a 6-well plate at a density of 4.0 × 10^5^ cells/mL for 24 h. The pNF-κB-*Luc* reporter plasmid and the pRL-TK plasmid were transfected into RAW 264.7 cells using Lipofectamine according to the manufacturer’s instructions. After transfection, the cells were treated with various concentrations of RAME (12.5, 25 and 50 μM) for 2 h and were subsequently incubated with LPS (1 μg/mL) for an additional 24 h. The cells were collected, and a dual-luciferase reporter assay system was used. The results are presented as means ± SD of three replicates for one representative experiment.

### 4.10. Measurement of IFN-β and MCP-1 Levels

The cells were cultured in a 6-well plate at a density of 2.0 × 10^5^ cells/mL for 24 h. After incubation, the cells were pretreated with various concentrations of RAME (12.5, 25 and 50 μM) for 2 h and then incubated with LPS (1 μg/mL) for an additional 4 h for measured IFN-β. RAW 264.7 cells were pretreated with RAME for prior to the addition of IFN-β (100 unit/mL) and incubated for an additional 8 h for measured MCP-1. The levels of IFN-β and MCP-1 proteins in the cell supernatant were measured using an ELISA kit according to the manufacturer’s protocol. The results are presented as means ± SD of three replicates for one representative experiment.

### 4.11. Statistical Analysis 

All data are presented as means ± SD. The differences in means between the treated and untreated groups were determined using the Student’s *t* test. A *p* value < 0.05 was considered to indicate statistical significance. # *p* < 0.05 vs. control group, * *p* < 0.05 vs. LPS-treated group and ** *p* < 0.05 vs. LPS + RAME group.

## 5. Conclusions

In this study, we investigated the underlying mechanism underlying the anti-inflammatory effects of RAME, isolated from the *P. frutescens* mutant, in RAW 264.7 cells. RAME inhibited NO production via suppression of iNOS expression, which was mediated by inhibition of NF-κB activation. It also inhibited the upstream and downstream pathways of IFN-β production. In addition, RAME induced HO-1 expression via Nrf2 activation, which contributed to its anti-inflammatory activity (Figure 7).

These results suggest that RAME inhibited NO production via both MyD88-dependent pathway and -independent pathways, as well as via the induction of HO-1 expression. RAME should be considered a potential anti-inflammatory agent for the treatment of inflammatory diseases.

## Figures and Tables

**Table 1:** Primer sequences used in quantitative real-time PCR.

Primers	Forward	Reverse
IL-1β	GAGAATGACCTGTTCTTTGAAGTTGAC	TGAAGCTGGATGCTCTCATCAG
IL-6	GTTCTCTGGGAAATCGTGGAA	GCAAGTCCATCATCGTTGTTC
IL-10	GACAACATACTGCTAACCGACTCC	TTCACCTGCTCCACTGCCTTG
HO-1	TTACCTTCCCGAACATCGAC	GCATAAATTCCCACTGCCAC
iNOS	TCCTACACACCAAACTGTGTGC	CTCCAATCTCTGCCTATCCGTCTC
β-actin	TGAGAGGGAAATCGTGCGTGAC	GCTCGTTGCCAATAGTGATGACC

Gene expression was compared with that of β–actin and evaluated by the comparative CT threshold protocol using the Bio-Rad Genex-Gene Expression Macro software tool [45]. The results are presented as means ± SDs of three replicates for one representative experiment.

## Supplementary Materials

Supplementary materials can be accessed at: http://www.mdpi.com/1420-3049/21/8/1083/s1.
