# Supplementary material for: Rosmarinic Acid Methyl Ester Inhibits LPS-Induced NO Production via Suppression of MyD88- Dependent and -Independent Pathways and Induction of HO-1 in RAW 264.7 Cells
Source: Molecules. 2016 Aug 18;21(8):1083. doi: 10.3390/molecules21081083 (PMC6274143; doi:10.3390/molecules21081083)
Supplement: Supplementary file 1 [file molecules-21-01083-s001.pdf]

Supplementary Materials: Rosmarinic Acid Methyl Ester Inhibits LPS-Induced NO Production via Suppression of MyD88- Dependent and -Independent Pathways and Induction of HO-1 in RAW 264.7 Cells

Yangkang So, Seung Young Lee, Ah-Reum Han, Jin-Baek Kim, Hye Gwang Jeong and Chang Hyun Jin

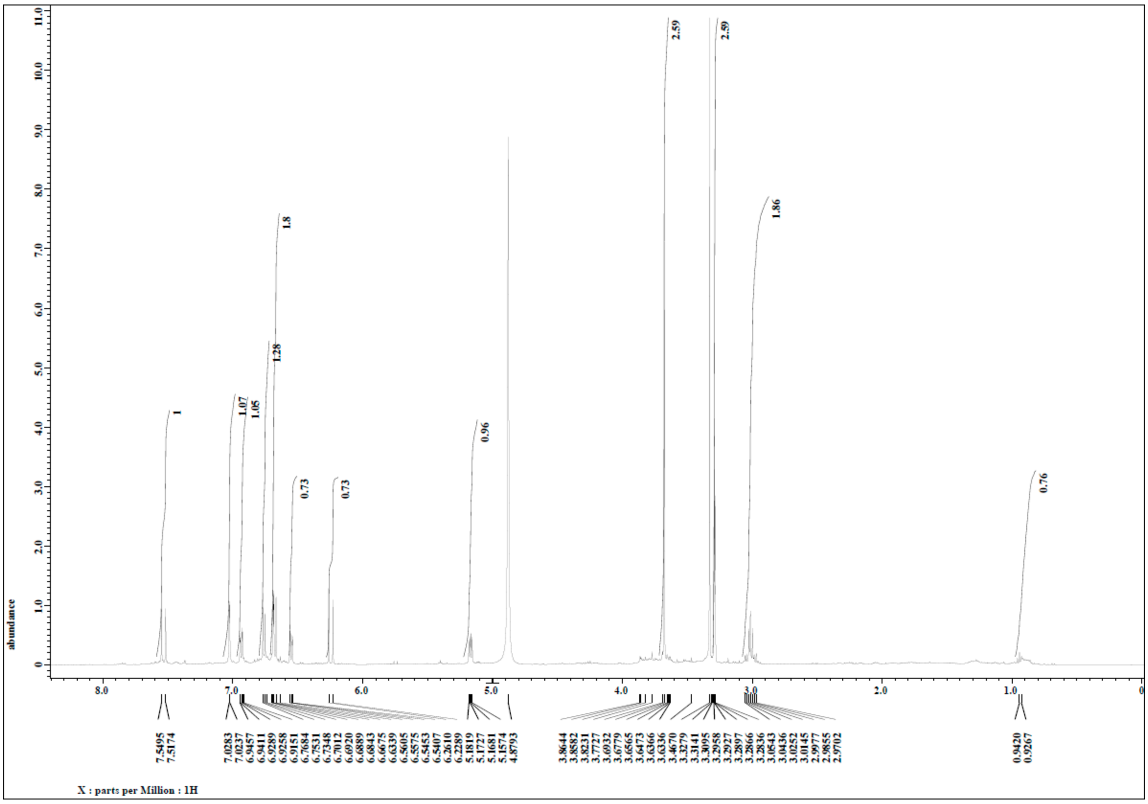

Figure S1. ^{1}H-NMR (500 MHz, CD_3OD) spectrum of rosmarinic acid methyl ester (RAME).
